# In vitro α-glucosidase inhibitory activity of isolated fractions from water extract of Qingzhuan dark tea

**DOI:** 10.1186/s12906-016-1361-0

**Published:** 2016-09-29

**Authors:** Shuyuan Liu, Zhi Yu, Hongkai Zhu, Wei Zhang, Yuqiong Chen

**Affiliations:** 1 Key Laboratory of Horticultural Plant Biology, Ministry of Education, College of Horticulture and Forestry Sciences, Huazhong Agricultural University, Wuhan, 430070 China; 2 College of Life Science, Xinyang Normal University, Xinyang, 464000 China

**Keywords:** *Camellia sinensis*, Dark tea, Tea extracts, α-Glucosidase, Anti-diabetes

## Abstract

**Background:**

Natural products have being used as potential inhibitors against carbohydrate-hydrolyzing enzymes to treat diabetes mellitus. Chinese dark tea has various interesting bioactivities. In this study, the active compounds from Qingzhuan dark tea were separated and their anti-diabetic activity was examined using an in vitro enzymatic model.

**Methods:**

The chloroform, ethyl acetate, *n*-butanol, sediment and residual aqua fractions of a Chinese dark tea (Qingzhuan tea) were prepared by successively isolating the water extract with different solvents and their in vitro inhibitory activities against α-glucosidase were assessed. The fraction with the highest inhibitory activity was further characterized to obtain the main active components of Qingzhuan tea.

**Results:**

The ethyl acetate fraction had the greatest inhibitory effect on α-glucosidase, followed by *n*-butanol, sediment and residual aqua fractions (with the IC_50_ values of 0.26 mg/mL, 2.94 mg/mL, 3.02 mg/mL, and 5.24 mg/mL, respectively), mainly due to the high content of polyphenols. Among the eight subfractions (QEF1-8) isolated from the ethyl acetate fraction, QEF8 fraction showed the highest α-glucosidase inhibitory potential in a competitive inhibitory manner (the *K*_*i*_ value of 77.10 μg/mL). HPLC-MS analysis revealed that (−)-epigallocatechin gallate (EGCG) and (−)-epicatechin gallate (ECG) were the predominant active components in QEF8.

**Conclusion:**

These results indicated that Qingzhuan tea extracts exerted potent inhibitory effects against α-glucosidase, EGCG and ECG were likely responsible for the inhibitory activity in Qingzhuan tea. Qingzhuan tea may be recommended as an oral antidiabetic diet.

## Background

Diabetes has become a major health problem in the world. It is a metabolic disease characterized by a high blood glucose level and can cause other health complications, such as cardiovascular disease, neuropathy, high blood pressure, weakness, gangrene, retinopathy, nephropathy and other dysfunctions [1]. One of the therapeutic approaches is focused on suppressing the glucose production from carbohydrates by inhibiting digestive enzymes, such as α-amylase and α-glucosidase [2, 3]. Acarbose has been used in the clinic therapy as an effective inhibitor of carbohydrate hydrolysis, but has adverse effects including abdominal pain, diarrhea and flatulence [4]. Effective and nontoxic inhibitors of α-amylase and α-glucosidase from natural products have been searched for the treatment of diabetes [5–7].

Tea (*Camellia sinensis*) is one of the most popular beverages worldwide possessing various pharmacological effects, such as anti-carcinogenic, hypoglycemic and anti-obesity activities [8–10]. Tea is divided into four types depending on the manufacturing process: non-fermented tea (green tea), semi-fermented tea (oolong tea), fermented tea (black tea) and post-fermented tea (dark tea) [11]. Dark tea, a post-fermented tea, is very popular in China, Mongolia, Russia and many other countries. The tea is made of mature fresh tea leaves through the processes of de-enzyming, rolling, pile-fermenting, drying, steaming, pressing and low temperature drying. During the long period of ‘pile-fermentation’, the chemical components change greatly to form the special characteristics, and the marker compounds are unclear so far.

Dark tea is not only an indispensable beverage but also a traditional Chinese medicine for the people in the northwest of China. Recently, it was reported that dark tea can inhibit cholesterol biosynthesis, protect against hydrogen peroxide, improve immune system and decrease inflammation [12]. Qingzhuan tea is one type of dark tea. Our previous investigations showed that Qingzhuan tea can exert anti-obesity and lipid-lowering effects in vivo [13, 14] and antioxidant and α-amylase inhibitory activities in vitro [15]. Here, we further report a study on the α-glucosidase inhibitory activity of Qingzhuan tea and its probable mechanism.

## Methods

### Materials

Tea samples (Qingzhuan tea) were obtained from Huanghelou Tea Factory (Hubei, China); α-glucosidase (EC 3.2.1.20 *Saccharomyces cerevisiae*, G0660) and *p*-nitrophenyl-α-D-glucopyranoside (*p*NPG, N1377) were purchased from Sigma-Aldrich (St. Louis, MO, USA). Catechins standards of (−)-epicatechin (EC), (+)-catechin (C), (−)-epigallocatechin (EGC), (−)-gallocatechin (GC), (−)-epicatechin gallate (ECG), (−)-catechin gallate (CG), (−)-epigallocatechin gallate (EGCG) and (−)-gallocatechin gallate (GCG) and gallic acid (GA) were purchased from Shanghai Yuanye Biological Technology Company Limited (Shanghai, China). Acarbose was acquired from the National Institute for the Control of Pharmaceutical and Biological Products (Beijing, China). All other reagents and solvents were purchased from China National Pharmaceutical Group Corporation (Beijing, China) and were of analytical grade. Purified water (18.2 MΩ) was prepared using a Millipore Mill-Q Ultrapure Water System (Billerica, MA, USA).

### Preparation of Qingzhuan tea extracts and subfraction of the ethyl acetate extract

Qingzhuan tea (50 g) was cut into thin slices and extracted with 1 L of boiling purified water for 7 min. The mixture was filtered through filter paper (Whatman 102, Hangzhou, China) under vacuum condition. The filtrate was collected and the residue was extracted once more under the same conditions. The two filtrates were combined and concentrated to 200 mL by the vacuum rotary evaporator (Jinye RE-52AA, Shanghai, China) at 55 °C under reduced pressure. The concentrated tea extracts (200 mL) were further extracted with 600 mL of chloroform, ethyl acetate and *n*-butanol separately three times to obtain the chloroform, ethyl acetate and *n*-butanol fractions. The aqua fraction was precipitated with a three-fold volume of 95 % ethanol (ethanol/water, 95:5 v/v) at room temperature for 24 h to obtain the sediment fraction and the residual aqua fraction. Each fractions was concentrated by vacuum rotary evaporation to remove the organic solvents completely and then freeze-dried (Labogene CoolSafe 110–4, Denmark).

Ethyl acetate fraction (0.2 g) was dissolved in a small amount of methanol and then loaded to a Sephadex LH-20 column (50 × 1.6 cm i.d. GE Healthcare Bio-Sciences AB, Sweden) and eluted successively with 500 mL of water, water/methanol (9:1 v/v, 8:2 v/v, 7:3 v/v, 6:4 v/v, 5:5 v/v, and 4:6 v/v respectively), and water/acetone (1:1 v/v) [15]. The isolated active components (QEF1-8) were concentrated by the vacuum rotary evaporator to remove the organic solvents completely and then further freeze-dried.

### Characterization of the Qingzhuan tea extracts and HPLC-MS analysis of QEF8 subfraction

Total polyphenols content in Qingzhuan tea extracts was determined according to the Folin-Ciocalteu method [16]. Water soluble carbohydrates were measured by the anthrone-sulfuric acid method [17]. HPLC analysis of caffeine was performed as described in our previous work [18] using an Agilent TC-C_18_ column (5 μm, 150 mm × 4.6 mm i.d.). The total theaflavins, thearubigins and theabrowins were measured by following the method of Zhong [19].

The QEF8 was analyzed by high performance liquid chromatography-mass spectrometry (HPLC-MS, Agilent 1100VL, USA). The HPLC parameters were set according to our previous work with slight modifications [18]. The sample was applied to an Agilent TC-C_18_ column (5 μm, 250 mm × 4.6 mm i.d.) with a gradient mobile phase consisted of water (0.1 % formic acid, A) and methanol (0.1 % formic acid, B) at a flow rate of 1.0 mL/min. A gradient elution was adopted as follows: 0–2 min, 80–75 % A; 2–6 min, 75–70 % A; 6–10 min, 70–75 % A; 10–13 min, 75–70 % A; 13–20 min, 70 % A; 20–23 min, 70–75 % A; 23–25 min, 75–80 % A; 25–30 min, 80 % A. The injection volume was 5 μL and detection wavelength was 278 nm. The temperature of column oven was set at 35 °C. The MS adopted a positive ion mode and scanned from 100 to 900 m/z. The flow rate of dry gas was 40 L/min at 250 °C. The capillary voltage, ESI voltage and discharge voltage were 3500 V, 10 kV, and 124.8 V, respectively. For the quantitative analysis, pure EC, C, EGC, ECG, CG, ECG, EGCG, GCG and GA were used as standards.

### Inhibitory assay of α-glucosidase

α-Glucosidase inhibitory activity was determined as described by Li et al. [20] with slight modifications. Briefly, mixtures of 112 μL potassium phosphate buffer (PBS, pH 6.8), 20 μL enzyme solution (1 unit/mL), and 8 μL of the sample (dissolved in DMSO) were incubated in a 96-well plate at 37 °C for 15 min, followed by the addition of 20 μL of *p*NPG (2.5 mmol/L) to each well, and incubation at 37 °C for 15 min. The reaction was terminated by adding 80 μL of Na_2_CO_3_ solution (0.2 mol/L). Absorbance was determined at 405 nm using a microplate reader (Bio-Tek ELX-800, USA). The control and blank were added 8 μL DMSO instead of the sample solution. Acarbose was used as a positive control. The α-glucosidase inhibitory activity was expressed as the IC_50_ according to the percentage inhibition and calculated by the following equation:

1 $$ \mathrm{Inhibitory}\;\mathrm{activity}\;\left(\%\right)=\left[1-\left(O{D}_{sample}-O{D}_{sample blank}\right)/\left(O{D}_{control}-O{D}_{blank}\right)\right]\times 100 $$

where OD_sample_ was the absorbance of PBS + enzyme + sample + *p*NPG; OD_sampleblank_ was the absorbance of PBS + sample + *p*NPG; OD_control_was the absorbance of PBS + enzyme + *p*NPG; OD_blank_ was the absorbance of PBS + *p*NPG. IC_50_: the half inhibition concentration of Qingzhuan tea fractions on α-glucosidase activity.

### Determination of the mode of α-glucosidase inhibition

The mixture of 20 μL *p*NPG substrate solution (at concentrations of 0.2 mmol/L, 0.4 mmol/L, 0.8 mmol/L, 1.0 mmol/L, 1.6 mmol/L, and 2.0 mmol/L), 112 μL PBS, and 8 μL sample (0, 20, 60 and 100 μg/mL) were incubated in a 96-well plate at 37 °C for 15 min, followed by the addition of 20 μL of the enzyme solution (1 unit/mL) to each well and 5 min incubation at 37 °C. The reaction was terminated by the addition of 80 μL of Na_2_CO_3_ solution (0.2 mol/L) and the OD values were measured at 405 nm. The concentration was calculated from the standard curve of the corresponding relationship between the OD values and the concentrations of product (*p*-nitrophenol, *p*NP). The *V*_*max*_ and *K*_*m*_constants were determined using the Lineweaver-Burk plots from the relevant Michaelis-Menten equations.

### Statistical analysis

Data were analyzed using SPSS statistical software (SPSS, Chicago, IL, USA). Values were expressed as mean ± standard deviation (SD) of three replications of the experiment. The differences in the mean values were analyzed using the Fisher’s least significant difference (LSD) procedure, and were considered significant at *p* < 0.05.

## Results and discussion

### α-Glucosidase inhibitory activity of Qingzhuan tea extracts

α-Glucosidase plays a central role in modulating postprandial hyperglycemia, which breaks down α-1,4-glucosidic linkages of disaccharides, resulting in simpler sugars. A previous study reported the established α-glucosidase inhibitors and their effects on delaying the expeditious generation of blood glucose after food uptake [21].

In the present study, the crude water extract of Qingzhuan tea was divided into five fractions by polarity and the α-glucosidase inhibitory activities of these fractions were detected using *p*NPG as the reaction substrate. As shown in Table 1, the crude water extract of Qingzhuan tea exerted an obvious inhibitory effect on α-glucosidase, with an IC_50_ value of 2.47 mg/mL. A previous in vivo study [22] indicated the hypoglycemic effect of dark tea for diabetic mice. In our experiment, no α-glucosidase inhibition was observed in the chloroform fraction of Qingzhuan tea, but the ethyl acetate, *n*-butanol, sediment and residual aqua fractions exhibited a dose-dependent inhibitory effect on α-glucosidase activity. The IC_50_ values of the four fractions ranged from 0.26 to 5.24 mg/mL, and followed the sequence of residual aqua fraction (5.24 mg/mL) > sediment fraction (3.02 mg/mL) > *n*-butanol fraction (2.94 mg/mL) > ethyl acetate fraction (0.26 mg/mL), indicating that the ethyl acetate fraction had the greatest inhibitory activity. Meanwhile, apart from the residual aqua fraction, the sediment, *n*-butanol and ethyl acetate fractions could exhibit significantly more potent inhibitory effects than acarbose (an effective inhibitor of α-glucosidase with the IC_50_ value of 4.64 mg/mL*, p* < 0.01), suggesting Qingzhuan tea extracts could be potential α-glucosidase inhibitors.

**Table 1:** Inhibitory effects of Qingzhuan tea fractions on α-glucosidase

Extracts	IC_50_ (mg/mL)	Regression equation	*R* ^*2*^
The crude water extract	2.47 ± 0.30 *	y = 0.120 × + 0.204	0.975
Chloroform fraction	No activity		
Ethyl acetate fraction	2.27 ± 0.03 *	y = 2.131 × − 0.057	0.984
*n-*Butanol fraction	2.94 ± 0.44 *	y = 0.171 × − 0.002	0.991
Sediment fraction	3.02 ± 0.07 *	y = 0.042 × + 0.373	0.991
Residual aqua fraction	5.24 ± 0.11	y = 0.101 × − 0.029	0.987
Acarbose	4.64 ± 0.57	y = 0.045 × + 0.291	0.987

*Note: n=*3, Mean ± SD

* *p* > 0.01 when compared to acarbose

In order to clarify the inhibitory mechanism of α-glucosidase activity, the basic active components in Qingzhuan tea extracts were determined (Table 2). The crude water extract contained 18.25 % polyphenols and 26.83 % tea-pigments (including theaflavins, thearubigins and theabrownins), 14.74 % carbohydrates, and 5.82 % caffeine. Due to the special pile-fermenting process, Qingzhuan tea had a high content of tea-pigments, especially thearubigins and theabrownins. Among the five fractions, the ethyl acetate fraction had the highest total polyphenols (62.72 %) and theaflavins (1.70 %), and the second highest amount of total thearubigins (24.21 %). Caffeine was the dominant active compound (70.55 %) in the chloroform fraction. The *n*-butanol fraction contained a certain amount of polyphenols (31.09 %), carbohydrates (15.56 %), thearubigins (28.00 %), and theabrownins (24.82 %). For the sediment fraction, 16.65 % carbohydrates, 9.16 % polyphenols, and 27.18 % theabrownins were obtained. The residual aqua fraction almost had the same components as the crude water extract.

**Table 2:** The main constituent of Qingzhuan tea extracts (%)

Extracts	Polyphenol	Carbohydrate	Caffeine	Theaflavin	Thearubigin	Theabrownin
The crude water extract	18.25 ± 0.21 cC	14.74 ± 1.02 bA	5.82 ± 0.06 bB	0.15 ± 0.01 cC	5.51 ± 0.14 bB	21.17 ± 0.21 bcC
Chloroform fraction	0.98 ± 0.06 fF	1.28 ± 0.33 dC	70.55 ± 0.01 aA	0.62 ± 0.00 bB	0.74 ± 0.00 cC	0.21 ± 0.00 eE
Ethyl acetate fraction	62.72 ± 2.63 aA	5.85 ± 1.16 cB	0.50 ± 0.01 cC	1.70 ± 0.23 aA	24.21 ± 3.18 aA	4.17 ± 0.17 dD
*n-*Butanol fraction	31.09 ± 0.73 bB	15.56 ± 0.07 abA	0.30 ± 0.002 dD	0.24 ± 0.03 cC	28.00 ± 1.68 aA	24.82 ± 1.25 abB
Sediment fraction	9.16 ± 0.35 eE	16.65 ± 0.04 aA	ND	0.02 ± 0.01 dD	ND	27.18 ± 0.80 aA
Residual aqua fraction	11.15 ± 0.31 dD	17.19 ± 0.20 aA	0.13 ± 0.02 eE	0.03 ± 0.03 dD	0.14 ± 0.19 cC	19.56 ± 0.05 cC

*Note:* At the same column, different capital letters mean a significant difference at *p* < 0.01 level and small letters at *p* < 0.05 level compare to each other, ND means not detected

All the aforementioned results indicated that the ethyl acetate fraction had the highest inhibitory effect on α-glucosidase, and the highest contents of total polyphenols and theaflavins. Correlation analysis between the levels of total polyphenols, theaflavins and IC_50_ values in ethyl acetate, *n*-butanol, sediment and residual aqua fractions showed that both polyphenols and theaflavins were tightly correlated with α-glucosidase inhibitory activities (*R*^*2*^ = −0.884 and −0.878, respectively), suggesting that α-glucosidase inhibitory activity was likely attributed to the synergistic interaction of polyphenols and theaflavins in the Qingzhuan tea extracts. It has been reported that plant phenolic compounds could inhibit α-glucosidase effectively [23]. Different teas (green, oolong and black teas) showed different α-glucosidase inhibitory profiles, which was associated with their major polyphenol contents [24]. Theaflavin was reported to have α-glucosidase inhibitory activity in the following order: theaflavin-3-O-gallate > theaflavin −3,3′-di-O-Gal > theaflavin −3′-O-Gal > theaflavin [25]. It seems that the presences of hydroxyl group and gallate group were closely associated with α-glucosidase inhibitory effects.

Polysaccharides have also been reported to have hypoglycemic activities, partly due to their α-glucosidase or α-amylase inhibitory activity [26, 27]. The present work also showed that the sediment fraction containing a substantial portion of polysaccharides exerted an ability to inhibit α-glucosidase. A similar result was observed that polysaccharides obtained from leaves and flowers of *Camellia sinensis* by different extraction methods have been reported to have α-glucosidase and α-amylase inhibitory activities [28].

The chemical components change greatly due to the special manufacturing process of Qingzhuan tea. For instance, catechins, the predominant polyphenols in tea, accounted for 70–80 % of total polyphenols [29] and could be oxidized and polymerized to generate theaflavins, thearubigins, theabrownins and even unidentified polyphenols polymers under fungal, damp and hot conditions during postfermentation [30]. Theabrownins were responsible for the characteristic color of Qingzhuan tea and were enriched in the sediment, *n*-butanol and residual aqua fractions. Although the chemical composition of theabrownins is still unknown, some biological activities have been demonstrated, such as serum lipid-lowering effect, antioxidant property and nitric oxide scavenging ability [31, 32]. It is possible that theabrownins also contribute to the α-glucosidase inhibition, which needs further research. Thearubigins have been reported to have α-glucosidase inhibitory activity, while the inhibitory effect was weaker than catechins and theaflavins [33]. Under the present isolation conditions, the *n*-butanol fraction had the highest thearubigins, which might also contribute to the α-glucosidase inhibitory activity.

### Inhibitory effect of eight subfractions from ethyl acetate fraction on in vitro α-glucosidase activity

The aforementioned results showed the ethyl acetate fraction had the greatest inhibitory effect on α-glucosidase activity in vitro, and thus it was further separated into eight subfractions on a Sephadex LH-20 column with different mixtures of methanol or acetone with water or water alone as mobile phase. The inhibitory effects of those subfractions on α-glucosidase were investigated, and their IC_50_ values were listed in Table 3. All subfractions showed α-glucosidase inhibition capabilities, and their IC_50_ values followed the sequence of QEF1 (12.31 mg/mL) > QEF2 (7.57 mg/mL) > QEF3 (6.22 mg/mL) > QEF4 (5.39 mg/mL) > QEF5 (3.33 mg/mL) > QEF6 (1.61 mg/mL) > QEF7 (0.95 mg/mL) > QEF8 (0.066 mg/mL), indicating that the QEF8 had the greatest inhibitory activity. As shown in Table 3, there was significant difference in the α-glucosidase inhibitory activities among the eight subfractions compared to acarbose (*p* < 0.01). The IC_50_ values of QEF5, QEF6, QEF7 and QEF8 were significantly lower than that of acarbose, especially QEF8, with IC_50_ value being 70-fold lower than that of acarbose and 4-fold lower than that of the ethyl acetate fraction.

**Table 3:** α-Glucosidase inhibition of various subfractions obtained from the ethyl acetate fraction

Subfraction	IC_50_ (mg/ML)	Regression equation	*R* ^*2*^
QEF1	12.31 ± 0.60 **	y = 0.074 × − 0.411	0.986
QEF2	7.57 ± 0.54 **	y = 0.075 × − 0.068	0.989
QEF3	6.22 ± 0.70 **	y = 0.076 × + 0.027	0.968
QEF4	5.39 ± 0.58	y = 0.079 × + 0.074	0.987
QEF5	3.33 ± 0.11 *	y = 0.157 × − 0.022	0.996
QEF6	1.61 ± 0.06 **	y = 0.200 × + 0.178	0.973
QEF7	0.95 ± 0.05**	y = 0.391 × + 0.128	0.961
QEF8	0.066 ± 0.01**	y = 4.640 × + 0.193	0.968
Acarbose	4.64 ± 0.57	y = 0.045 × + 0.291	0.987

*Note: n* = 3, Mean ± SD

* *p* < 0.05, ** *p* < 0.01 when compared to acarbose

**Fig. 1:**
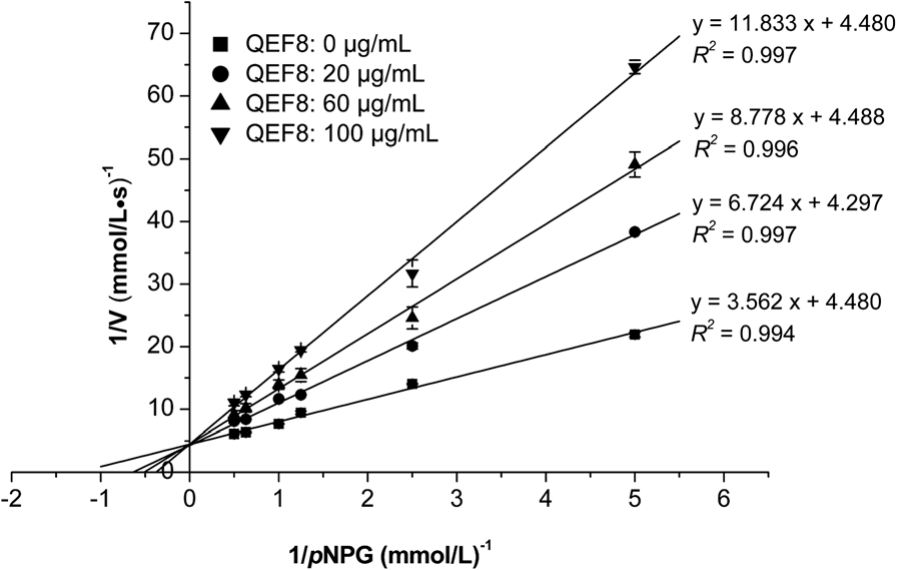
Lineweaver-Burk plot of QEF8 towards the substrate *p*NPG at different concentrations

The inhibitory mechanisms of QEF8 were further explored in this study. As shown in Fig. 1, the Lineweaver-Burk plots for various concentrations of QEF8 showed the same intersection on y-axis, indicating that the 1/*V*_*max*_ remained unchanged in the absence of the substrate. The QEF8 inhibited the enzymatic activity in a competitive manner. According to Michaelis-Menten equations, the value of the inhibition constant (*K*_*i*_) was 77.10 μg/mL (Table 4). Therefore, the mode of action of the inhibitory activity of QEF8 on α-glucosidase could be similar to that of acarbose. Acarbose has been shown in an animal study to be a competitive inhibitor of intestinal brush-border α-glucosidase enzymes through the acarviosine moiety binding with high affinity to the active centers of the proteins in this enzyme family [34].

**Table 4:** *K*_*m*_ and *V*_*max*_ values of α-glucosidase in the presence of different concentration of QEF8

Concentration of QEF8 (mg/mL)	*K*_*m*_ (mmol/L)	*V*_*max*_ (mmol/L s)	*K*_*i*_ (μg/mL)
0.00	0.81 ± 0.01 d	0.23 ± 0.005 a	77.10
0.02	1.54 ± 0.05 c	0.23 ± 0.007 a	
0.06	2.00 ± 0.08 b	0.220 ± 0.003 a	
0.10	2.64 ± 0.10 a	0.23 ± 0.006 a	

*Note:* At the same column, different small letters mean a significant difference at *p* < 0.05 level compared to each other

**Fig. 2:**
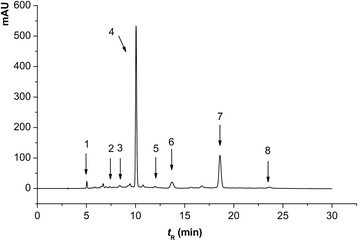
HPLC chromatogram of individual phenolic compounds in the QEF8. peak 1 GA: [M + H]^+^ 171.0, MS^2^ 152.8, 126.9; peak 2 EGC: [M + H]^+^ 307.1, MS^2^ 288.9, 138.9; peak 3 C: [M + H]^+^ 291.0, MS^2^ 273.4, 139.4; peak 4 EGCG: [M + H]^+^ 459.2, MS^2^ 288.9, 138.9; peak 5 GCG: [M + H]^+^ 459.2, MS^2^ 288.9, 138.9; peak 6 EC: [M + H]^+^ 291.0, MS^2^ 273.4, 139.4; peak 7 ECG: [M + H]^+^ 443.1, MS^2^ 272.9, 150.9; and peak 8 CG: [M + H]^+^ 443.1, MS^2^ 272.9, 150.9

**Fig. 3:**
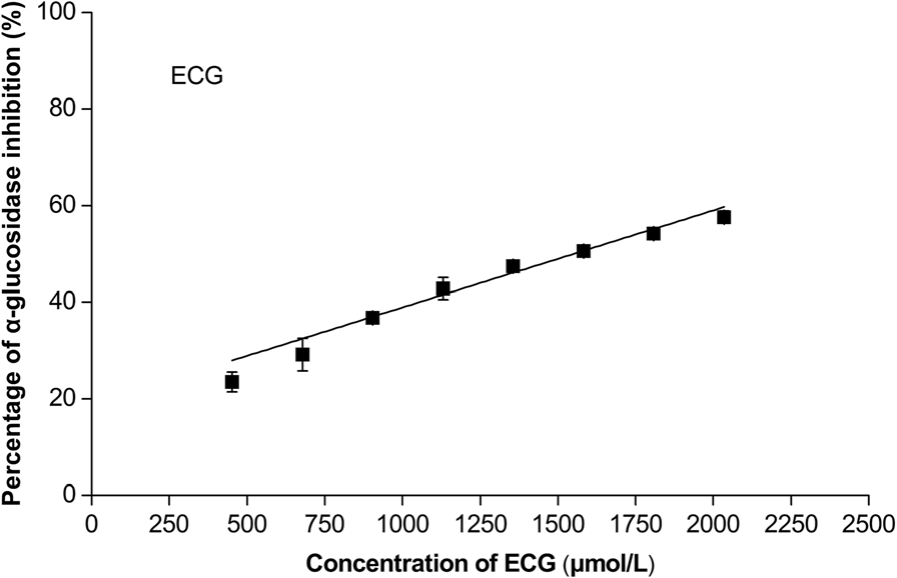
Inhibitory effects of ECG on α-glucosidase

**Fig. 4:**
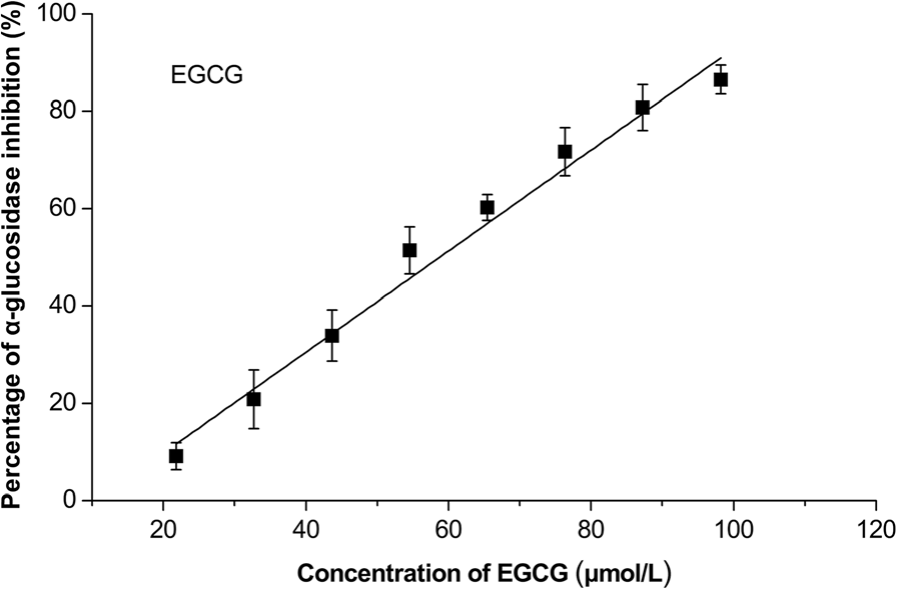
Inhibitory effects of EGCG on α-glucosidase

The components in QEF8 were analyzed by HPLC-MS method (Fig. 2). EGCG (m/z 459.2) and ECG (m/z 443.1) were found to be the predominant active compounds in QEF8 (Table 5). To further confirm which components in QEF8 are the most effective as inhibitors, EGCG and ECG were tested on the inhibition of α-glucosidase. As shown in Figs. 3 and 4, EGCG showed greater inhibitory effect than ECG, with IC_50_ values of 59 μmol/L (27.02 μg/mL) and 1626 μmol/L (719.29 μg/mL), respectively. Matsui et al. [25] investigated the α-glucosidase inhibitory activities of catechins (EC, ECG, EGC and EGCG) and found EGCG exhibited greater inhibitory activity of maltase than ECG in rat intestinal acetone powder. Kamiyama et al. [35] further compared the inhibitory activities of ten catechins toward maltase and sucrase in rat brush border membrane vesicles prepared freshly and obtained a similar result. Those results showed that galloylated catechins had higher α-glucosidase inhibitory activities than non-galloylated catechins. The galloy group bonding at the 3 position of catechins played an important role in the α-glucosidase inhibitory activity. Among galloylated catechins, the number of the hydroxyl group on the B ring was favorable to the inhibitory activity. Galloylated catechins also had higher inhibitory effects on α-amylase that was another important digestive enzyme, while catechol catechins (CG and ECG) were 2 times more inhibitory than pyrogallol catechins (GCG and EGCG) [36]. Intestinal glucose transporters were responsible for subsequent glucose uptake, and ECG was also more effective against intestinal glucose transport than EGCG [37, 38]. Xu et al. [39] assessed the contribution of seven catechins to the inhibition of carbohydrate digestive enzyme (α-glucosidase and α-amylase) and intestinal glucose transport, and found the inhibitory potency of seven catechins was ranked in a similar order. The inhibitory kinetics of catechins on α-glucosidase has been reported and still remain controversial. Li et al. [40] reported that both EGCG and ECG inhibited *Saccharomyces cerevisiae* α-glucosidase in non-competitive manners. While the mode of rat intestinal α-glucosidase of EGCG was a mix-competitive manner and the *K*_*i*_ value was 87.8 μg/mL in the study of Xu et al. [39].

**Table 5:** Summary of data used to quantify major components of QEF8 by HPLC method

Compound	Regression equation	*R* ^*2*^	Linear range (μg/mL)	LOD (μg/mL)	Content (%)
GA	y = 12.285 × − 8.215	0.9997	3.91–31.25	0.76	0.60 ± 0.03
EGC	y = 1.112 × − 8.101	0.9998	54.69–437.50	9.59	0.55 ± 0.23
C	y = 2.214 × − 2.175	0.9998	9.38–75.00	1.34	1.29 ± 0.15
EGCG	y = 7.721 × − 107.250	0.9989	62.50–500.00	24.05	30.94 ± 0.45
GCG	y = 1.530 × − 4.051	0.9999	25.00–200.00	3.42	2.74 ± 0.20
EC	y = 20.066 × − 33.854	0.9987	9.38–75.00	3.84	1.18 ± 0.02
ECG	y = 9.056 × − 30.433	0.9995	18.75–150.00	4.9	10.61 ± 0.15
CG	y = 8.725 × − 8.939	0.9993	4.69–37.50	1.40	0.53 ± 0.04

*Note:* LOD (limit of detection) was defined as the value equal to a signal-to-noise ratio of three

Tea has been associated with reducing the risk of diabetes, while the ability of various teas on antidiabetic effects was different. Koh et al. [41] investigated the ability of different teas (green, oolong and black teas) in inhibiting α-glucosidase and α-amylase, and found black tea was most potent in inhibiting α-glucosidase and α-amylase. Theaflavins in black tea were associated with their potent inhibitory effects, and catechins were weaker enzyme inhibitors in contrast to theaflavins. In the study of Yang et al. [24], the inhibitory effect of oolong tea extract was significantly higher than that of green tea and black tea extracts. The difference might be due to different sources of tea, which correlated to their major polyphenol content (theaflavins and catechins). Gomes et al. [42] revealed that black tea extract was more effective in reducing the blood glucose concentration in streptozotocin-induced diabetic rats comparing with green tea extract, while green tea extract was more effective in preventive. Tang et al. [43] performed the comparison between green tea extract and black tea extract in a type II diabetic mouse model, suggesting that different tea extracts might exert hypoglycaemic effects through different pathways. Qingzhuan tea, as a post-fermentation tea, might reduce blood glucose in a different way. Our previous investigation showed that Qingzhuan tea was more effective than green tea in losing weight, decreasing serum lipids, antioxidation and protecting liver cells of hypolipidemic rats [14]. Cheng et al. [15] tried to identify bioactive components from Qingzhuan tea by successively isolating the water extract and found QEF8 was the most active subfraction based on the in vitro antioxidant and α-amylase inhibitory activity, which is consistent with the present results. HPLC chromatographic separation of QEF8 revealed that almost half of the subfraction was catechins. It can concluded that catechins were an important factor in contributing to the antioxidant and digestive enzymes inhibitory activity of Qingzhuan tea. While during the process of Qingzhuan tea, the post-fermentation stage process decreased the contents of catechins and formed some novel catechins derivatives. These unidentified derivatives probably contributed to the anti-hyperglycemic potential, which still requires further research.

## Conclusion

α-Glucosidase plays an important role in carbohydrate digestion and is considered as one of the targets for regulating postprandial hyperglycemia in the therapy of diabetes type II. The results from this study indicated that Qingzhuan tea extracts exerted potent inhibitory effects against α-glucosidase, and EGCG and ECG were found to be responsible for the inhibitory activity, implying the potential of Qingzhuan tea as an oral antidiabetic diet.

## Abbreviations

C: (+)-catechin
CG: (−)-catechin gallate
EC: (−)-epicatechin
ECG: (−)-epicatechin gallate
EGC: (−)-epigallocatechin
EGCG: (−)-epigallocatechin gallate
GA: Gallic acid
GC: (−)-gallocatechin
GCG: (−)-gallocatechin gallate
HPLC-MS: High performance liquid chromatography-mass spectrometry
LOD: Limit of detection
LSD: The Fisher’s least significant difference
PBS: Potassium phosphate buffer
*p*NP: *p*-nitrophenol
*p*NPG: *p*-nitrophenyl-α-D-glucopyranoside
QEF1-8: The eight subfractions isolated from the ethyl acetate fraction
SD: Standard deviation
